# Comparative Analysis of Inhaled Insulin With Other Types in Type 1 Diabetes Mellitus: A Systematic Review and Meta-Analysis

**DOI:** 10.7759/cureus.23731

**Published:** 2022-04-01

**Authors:** Abu baker Khan, Aftab Ahmad, Saad Ahmad, Maryam Gul, Fatima Iqbal, Hazrat Ullah, Syeda Laiba, Umer Khayaam Orakzai

**Affiliations:** 1 Surgery, District Headquarter Hospital, Dera Ismail Khan, PAK; 2 Internal Medicine, Category D Hospital, South Waziristan, PAK; 3 Internal Medicine, Taj Medical Center, Nowshera, PAK; 4 Medicine, THQ Hospital Ghilgo, Hungu, PAK; 5 Medicine, Khalifa Gulnawaz Teaching Hospital, Bannu, PAK; 6 Medicine, THQ Hospital Ghiljo, Hangu, PAK

**Keywords:** comparative study, hypoglycemic shift, subcutaneous insulin, type 1 diabetes mellitus, inhaled insulin

## Abstract

To analyze the effect of Inhaled insulin in Type 1 Diabetes Mellitus and compare it with other routes of administration of Insulin. A systemic search was conducted from the following electronic databases: PubMed/Medline, Cochrane Library, and Google Scholar, from inception to 28th January 2022. All statistical analysis was conducted in Review Manager 5.4.1. All studies meeting inclusion criteria were selected. A random-effect model was used to pool the studies, and the result was reported in the Standard Mean Difference (SMD), Mean Difference (MD), and Risk Ratio (RR) with their corresponding 95% Confidence interval (CI). Thirteen randomized control trials were selected for our meta-analysis. Statistically significant results were obtained for comparing change in weight after insulin administration (MD= -1.08 [-1.21, -0.94]; p< 0.00001; I2= 74%). Other factors assessed were found to be non-significant like HbA1c (SMD= 0.03 [-0.80, 0.86]; p= 0.95; I2= 99%), fasting blood sugar (SMD= -0.31 [-1.52, 0.91]; p= 0.62; I2= 99%) and adverse effects (RR= 1.06 [0.97, 1.16]; p= 0.18; I2= 96%). In this systematic review and meta-analysis, we found that inhaled insulin is equally effective as subcutaneously administered insulin in patients with Type 1 Diabetes. The inhaled insulin was found to show less weight gain and fewer hypoglycemic shifts, with a similar effect on the blood glucose level. No significant difference was observed in the incidence of adverse events.

## Introduction and background

Type 1 Diabetes (T1D) is an autoimmune disorder with an absolute deficiency of insulin, characterized by the destruction of pancreatic beta cells. It has a prevalence of 500,000 children worldwide [1]. T1D accounts for 5-10% of all cases of diabetes. Type 2 diabetes is the most common form of diabetes in adults [2]. Individuals with a positive family history, autoimmune disease, and HLA associations are highly susceptible to T1D [3]. The most common symptoms of T1D patients include polyuria (96%), weight loss (61%), and fatigue (52%) [4]. It is diagnosed with a fasting blood glucose test above 7.0 mmol/L, or a random blood glucose test above 11.1 mmol/L [5]. 

Several treatment regimens are used to manage T1D patients that include mealtime daily injections of rapid-acting insulin combined with daily basal insulin as well as continuous subcutaneous insulin infusion [3, 6]. Regular insulin (neutral insulin) is made using recombinant DNA technology. It is injected subcutaneously and has a slightly delayed onset of action, taken 15-30 mins before mealtime [6]. Neutral Protamine Hagedorn (NPH) insulin is formed by the addition of zinc to regular insulin and is injected subcutaneously. It has a variable action profile, with durations of action of about 14 hours [7]. Insulin glargine is a long-acting insulin that forms precipitates when injected subcutaneously, and it has a duration of action of 22-24 hours [7]. Rapid-acting analogs like insulin lispro, aspart, and glulisine are injected subcutaneously or intravenously, have a rapid onset (5-30 mins), and short duration of action (3-5 hrs) [8]. 

Inhaled insulin (technosphere) is powdered insulin formulated by adding fumaryl diketopiperazine (FDKP) to regular insulin [8]. It quickly dissolves upon inhalation, reaching a maximum (Tmax) concentration in about 15 minutes of inhalation as compared to rapid-acting insulin which has a Tmax of about 40 minutes [8-10]. Doses range from 10 to 80 units using the Gen2 inhaler device, and it has a bioavailability of 21% to 30% compared with regular insulin [9]. In this meta-analysis, we evaluated the effectiveness and adverse effects of inhaled insulin, in comparison to subcutaneous insulin injections.

## Review

Method 

Data Sources and Search Strategy

This systematic review and meta-analysis was conducted according to the Preferred Reporting Items for Systematic Review and Meta analyses (PRISMA) guidelines [11, 12]. An electronic search from PubMed/Medline, Cochrane Library, and Google Scholar was conducted from their inception to 28th January 2022 using the following search string: (Effectiveness OR efficacy OR safety OR application) AND (Insulin OR exubera) AND (Inhaled). In addition, we manually screened the cited articles of previous meta-analyses, cohort studies and review articles to identify any relevant studies. 

Study Selection

All studies were included if they met the following eligibility described as PICOS: 1) P (Population): Type 1 Diabetes Mellitus; 2) I (Interventions): Inhaled insulin; 3) C (Control): Insulin route other than inhaled; 4) O (Outcome): comparison between inhaled insulin and other routes of administration of insulin; 5) S (Studies): Human-based Randomized controlled trials published in English only. 

Cohort, case series, case reports, literature reviews, editorials, and studies not meeting the inclusion criteria were excluded. 

Data Extraction and Quality Assessment of Studies

Two reviewers independently searched electronic databases. Studies searched were exported to the EndNote Reference Library software version 20.0.1 (Clarivate Analytics), and duplicates were screened and removed.       

Data extraction and quality assessment of included studies were done simultaneously and independently by two reviewers. The Cochrane Collaboration’s Tool for randomized controlled trials was used to assess the quality of published trials (details are provided in Table 1). 

**Table 1:** Quality assessment using Cochrane Collaboration’s Tool

Study	Random sequence generation	Allocation concealment	Blinding (participants and personnel)	Blinding (outcome assessment)	Incomplete outcome data	Selective reporting	Other sources of bias	Net Risk of bias
Skyler et al., 2001 [13]	Low Risk	Unclear Risk	High Risk	Unclear Risk	Low Risk	Low Risk	Low Risk	Low Risk
Quattrin et al., 2004 [14]	Low Risk	Unclear Risk	High Risk	Unclear Risk	Low Risk	Low Risk	Low Risk	Low Risk
Skylar et al., 2005 [15]	Low Risk	Unclear Risk	High Risk	Unclear Risk	Low Risk	Low Risk	Low Risk	Low Risk
Garg et al., 2006 [16]	Low Risk	Unclear Risk	High Risk	Unclear Risk	Low Risk	Low Risk	Low Risk	Low Risk
Skylar et al., 2007 [17]	Low Risk	Unclear Risk	High Risk	Unclear Risk	Low Risk	Low Risk	Low Risk	Low Risk
Skyler et al., 2008 [18]	Low Risk	Unclear Risk	High Risk	Unclear Risk	Low Risk	Low Risk	Low Risk	Low Risk
White et al., 2008 [19]	Low Risk	Unclear Risk	High Risk	Unclear Risk	Low Risk	Low Risk	Low Risk	Low Risk
Comulada et al., 2009 [20]	Low Risk	Unclear Risk	High Risk	Low Risk	Low Risk	Low Risk	Low Risk	Low Risk
Garg et al., 2009 [21]	Low Risk	Low Risk	High Risk	Low Risk	Low Risk	Low Risk	Low Risk	Low Risk
Moses et al., 2009 [22]	Low Risk	Unclear Risk	High Risk	Unclear Risk	Low Risk	Low Risk	Low Risk	Low Risk
Bode et al., 2015 [23]	Low Risk	Unclear Risk	High Risk	Unclear Risk	Low Risk	Low Risk	Low Risk	Low Risk
Seaquist et al., 2020 [24]	Low Risk	Unclear Risk	High Risk	Unclear Risk	Low Risk	Low Risk	Low Risk	Low Risk
Mcgill et al., 2021 [25]	Low Risk	Unclear Risk	High Risk	Unclear Risk	Low Risk	Low Risk	Low Risk	Low Risk

Statistical Analysis

Review Manager (version 5.4.1; Copenhagen: The Nordic Cochrane Centre, The Cochrane Collaboration, 2020) was used for all statistical analyses. The data from studies were pooled using a random-effects model. Analysis of results was done by calculating the Standard Mean Difference (SMD), Mean Difference (MD) and Risk Ratio (RR) with respective 95% confidence intervals (CI). The chi-square test was performed to assess any differences between the subgroups. Sensitivity analysis was done to see if any individual study was driving the results and to implore reasons of high heterogeneity. As per Higgins et al, scale for heterogeneity was considered as follows: I2 = 25-60% - moderate; 50-90% - substantial; 75-100% - considerable heterogeneity, and p< 0.1 indicated significant heterogeneity [12]. A p< 0.05 was considered significant for all analyses. 

Results 

Literature Search Results 

**Figure 1:**
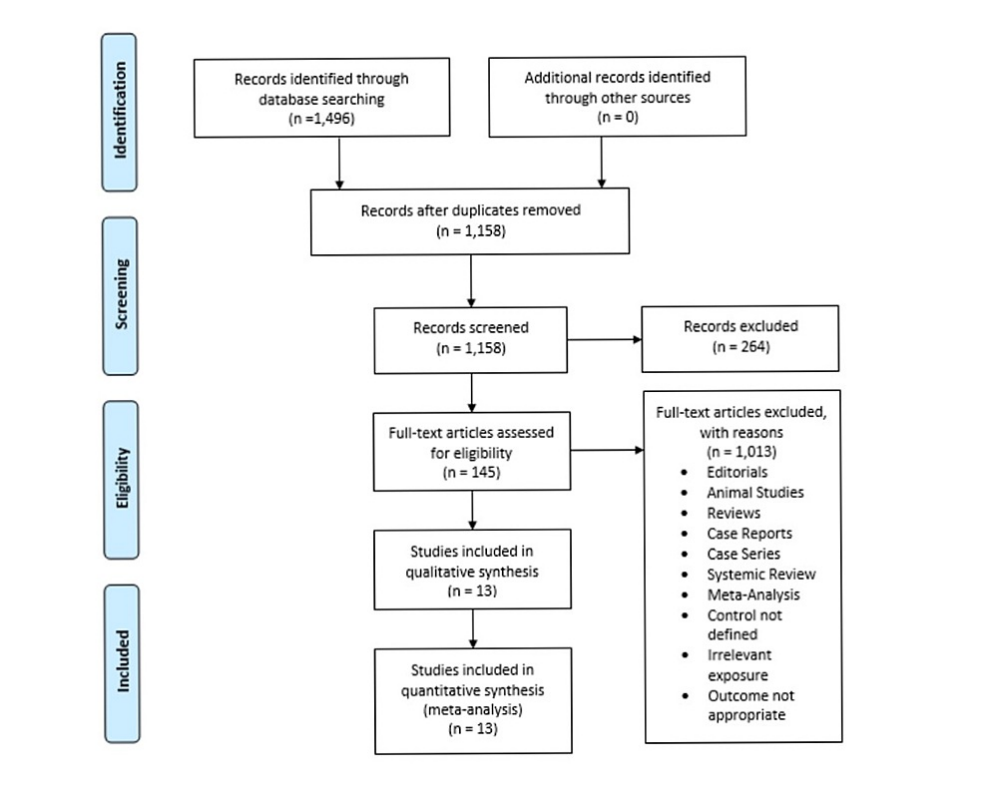
Prisma Flow Chart

The initial search of the three electronic databases yielded 1,496 potential studies. After exclusions based on titles and abstracts, the full texts of 145 studies were read for possible inclusion.  A total of 13 studies remained for quantitative analysis. Figure 1 summarizes the results of our literature search. 

Study Characteristics

Table 2 provides the basic characteristics of included studies [13-25]. Our analysis included 13 published studies. All studies are Randomized Controlled Trials. There are 3,899 patients in our analysis. The studies were conducted in different regions of the world i.e USA, Canada, Argentina, Mexico, Brazil, France, Australia, Argentina, Belgium, Italy, Puerto Rico, Russia and Ukraine. The mean age of patients was 34.34 years. 

**Table 2:** Basic Characteristics of selected articles

Study	Year	Study design	Duration	Country	BMI (kg/m2)	Total patients (n)		Male (%)		Mean Age (years)	Type of Insulin and Route (Experimental)	Type of Insulin and Route (Control)	Risk of Bias
Skyler et al., 2001 [13]	2001	RCT	N/A*	USA	25.02	72	52.7		37.5		Insulin (inhaled) + Ultralente Insulin (subcutaneous)	Usual Insulin regimen (injected)	Low Risk
Quattrin et al., 2004 [14]	2004	RCT	N/A*	USA, and Canada	25.07	334	53.5		33.7		Exubera Insulin (inhaled) + ultralente (subcutaneous)	NPH insulin or Insulin Glargine (subcutaneous)	Low Risk
Skylar et al., 2005 [15]	2005	RCT	N/A*	USA, and Canada	24.35	327	53.2		29.5		Exubera Insulin (inhaled) + NPH Insulin (subcutaneous)	Regular Insulin (subcutaneous) + NPH Insulin (subcutaneous)	Low Risk
Garg et al., 2006 [16]	2006	RCT	N/A*	USA	28.1	137	46.7		39		12 weeks of Human inhaled Insulin powder (inhaled) + Insulin Glargine (subcutaneous) followed by 12 weeks of Human Insulin or Insulin lispro (subcutaneous) + Insulin Glargine (subcutaneous)	12 weeks of Human Insulin or Insulin lispro (subcutaneous) + Insulin Glargine (subcutaneous) followed by 12 weeks of Human Inhaled insulin powder (inhaled) + Insulin Glargine	Low Risk
Skylar et al., 2007 [17]	2007	RCT	N/A*	USA, Canada, Argentina, Mexico, and Brazil	25.05	580	57		37.05		Exubera Insulin (inhaled) + ultralente or NPH Insulin or Insulin Glargine (subcutaneous)	Regular Insulin, Insulin lispro, or Insulin aspart (subcutaneous) + Ultralente or NPH Insulin or Insulin Glargine (subcutaneous)	Low Risk
Skyler et al., 2008 [18]	2008	RCT	N/A*	USA, Canada, Argentina, Mexico, and Brazil	N/A*	330	N/A*		N/A*		Exubera Insulin (inhaled) + ultralente or NPH Insulin or Insulin Glargine (subcutaneous)	Regular Insulin, Insulin lispro, or Insulin aspart (subcutaneous) + ultralente or NPH Insulin or Insulin Glargine (subcutaneous)	Low Risk
White et al., 2008 [19]	2008	RCT	N/A*	USA	19.15	120	N/A*		8.85		Exubera Insulin (inhaled) + Ultralente or NPH Insulin (subcutaneous)	Regular Insulin or Insulin lispro (subcutaneous) + Ultralente or NPH Insulin (subcutaneous)	Low Risk
Comulada et al., 2009 [20]	2009	RCT	N/A*	USA, India, Mexico, France, Germany, Argentina, Belgium, Italy, and Puerto Rico	25.23	500	57.02		39.25		AIR Insulin (inhaled) + Insulin Glargine (subcutaneous)	Insulin lispro (subcutaneous) + Insulin Glargine (subcutaneous)	Low Risk
Garg et al., 2009 [21]	2009	RCT	N/A*	USA, India, Belgium, Hungary, Canada, and Croatia	25.85	385	58		39.2		AIR Insulin (inhaled) + Insulin Glargine (subcutaneous)	Human Insulin or Insulin lispro (subcutaneous) + Insulin Glargine (subcutaneous)	Low Risk
Moses et al., 2009 [22]	2009	RCT	N/A*	Australia	26.4	299	55.2		40.5		Human Insulin (inhaled) + NPH Insulin (injected)	Insulin Aspart (subcutaneous) + NPH Insulin (injected)	Low Risk
Bode et al., 2015 [23]	2015	RCT	Feb 2011- May 2013	USA, Russia, Ukraine, and Brazil	N/A*	344	N/A*		N/A*		Technosphere Insulin (inhaled)	Insulin Aspart (injected)	Low Risk
Seaquist et al., 2020 [24]	2020	RCT	N/A*	USA	N/A*	345	N/A*		N/A*		Technosphere Insulin (inhaled) + basal Insulin (Insulin glargine, Insulin detemir, or NPH)	Insulin Aspart (subcutaneous) + basal Insulin (Insulin glargine, Insulin detemir, or NPH)	Low Risk
Mcgill et al., 2021 [25]	2021	RCT	N/A*	USA	25.4	126	57.35		38.85		Technosphere Insulin (inhaled) + Insulin Glargine (subcutaneous)	Insulin Lispro (subcutaneous) + Insulin Glargine (subcutaneous)	Low Risk

Publication Bias and Quality Assessment

**Figure 2:**
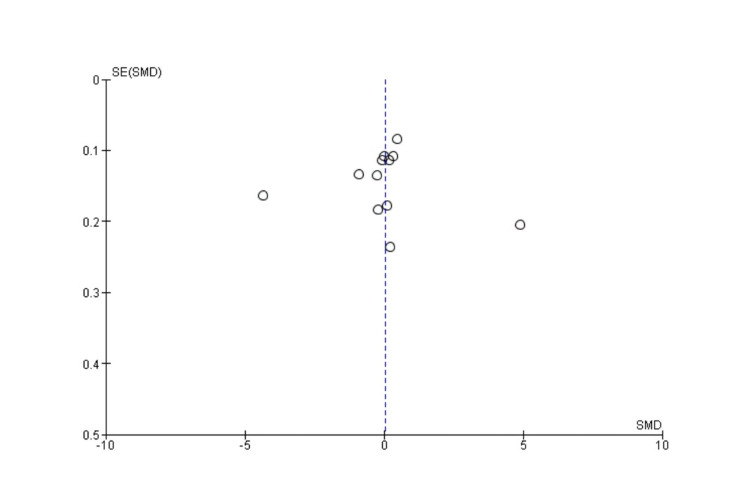
Funnel plot to assess publication bias

The visual inspection of the funnel plot (Figure 2) did not indicate that there is publication bias in our meta-analysis. 

All studies have low risk of bias (Table 2). 

Results of Meta-Analysis

**Figure 3:**
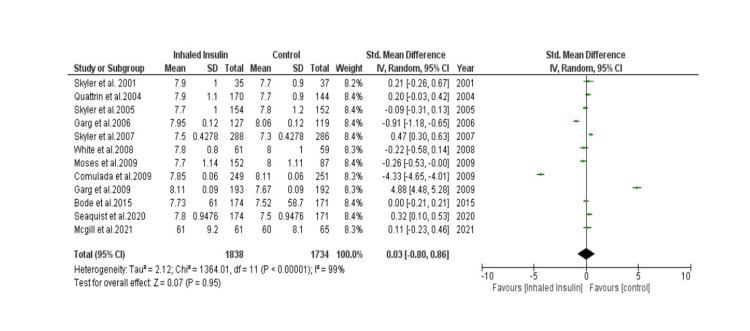
Forest plot showing effect size of Inhaled Insulin vs Control in HbA1c

**Figure 4:**
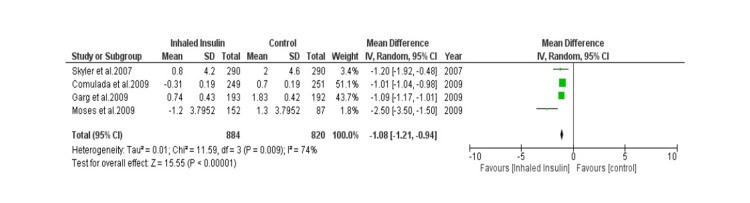
Forest plot showing effect size of Inhaled Insulin vs Control in Change in weight

**Figure 5:**
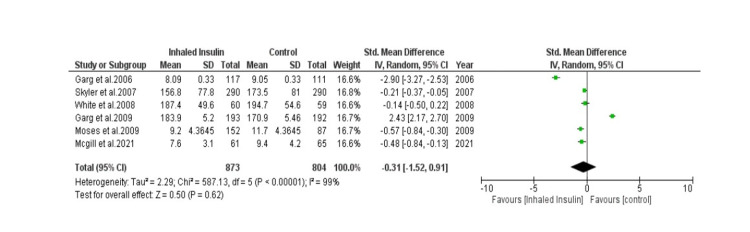
Forest plot showing effect size of Inhaled Insulin vs Control in Fasting Blood Glucose

**Figure 6:**
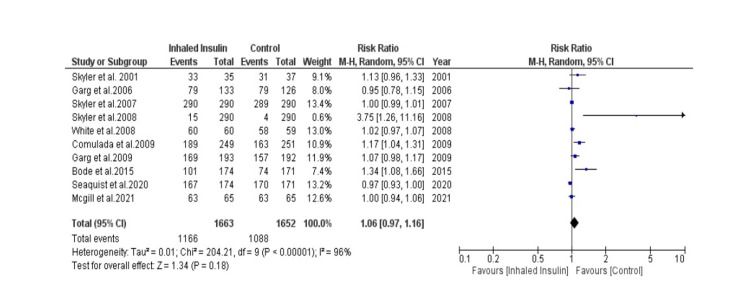
Forest plot showing effect size of Inhaled Insulin vs Control in Adverse events

Detailed forest plots, outlining the effect size of Inhaled insulin in HbA1c (Figure 3), Change in body weight (Figure 4), fasting blood glucose (Figure 5) and its adverse effects (Figure 6) as compared to the controls, are provided in the manuscript.

HbA1c: Out of thirteen studies, twelve studies were selected to analyze HbA1c [13-17, 19-25]. A total of 1,838 patients were in experimental group while 1,734 patients were in control group. Pooled result (Figure 3) showed that there was statistically non-significant Standard Mean Difference between the two groups (SMD= 0.03 [-0.80, 0.86]; p= 0.95; I2= 99%). 

Change in body weight: Out of thirteen studies, four studies provided data to analyze change in body weight [17, 20-22]. A total of 884 patients were used in experimental group while 820 patients were used in control group. Pooled result (Figure 4) showed that there was statistically significant Mean Difference which showed decrease change in weight in patients using inhaled insulin (MD= -1.08 [-1.21, -0.94]; p< 0.00001; I2= 74%). 

Fasting blood glucose: Out of thirteen studies, six studies were used to analyze fasting blood glucose [16, 17, 19, 21, 22, 25]. A total of 873 patients were present in experimental group while 804 patients were present in control group. Pooled result (Figure 5) showed that there was statistically non-significant effect of both interventions in fasting blood glucose (SMD= -0.31 [-1.52, 0.91]; p= 0.62; I2= 99%). 

Adverse effects: Out of thirteen studies, ten studies were used to identify adverse effects [14, 15, 22]. A total of 1,663 patients were there in experimental group while 1,652 patients were there in control group. Pooled result (Figure 6) showed that there was statistically non-significant risk ratio compared between the two groups (RR= 1.06 [0.97, 1.16]; p= 0.18; I2= 96%). 

Sensitivity Analysis 

A sensitivity analysis was conducted to assess the influence of each study on the overall effect by excluding one study at a time, followed by the generation of pooled Standard Mean Difference (SMD), Mean Difference (MD) and Risk Ratio (RR) for the rest of the studies. No significant change was observed after the exclusion of any individual study, suggesting the results were robust. 

Discussion 

In this systematic review and meta-analysis, we presented the assessment of evidence from thirteen randomized control trials, to evaluate the effectiveness of inhaled insulin in comparison to conventional insulin in patients diagnosed with type 1 diabetes. The results of our analysis showed insignificant improvement in HbA1c with respect to control, similar effects were observed in terms of fasting blood glucose and adverse events. However, inhaled insulin was associated with less weight gain than conventional insulin.  

Ceglia et al. in 2006, evaluated the effectiveness of inhaled insulin with subcutaneous insulin, and found that the inhaled premeal insulin administration was associated with glycemic efficacy less than that of subcutaneous insulin [26]. Pittas et al. in 2015 published a meta-analysis on this subject. The results of their study suggest that the glycemic efficacy of Technosphere insulin was lower than that of subcutaneous insulin, however, the inhaled insulin was found to have beneficial effects on body weight and was not associated with severe hypoglycemia [27]. None of the previously published meta-analyses accounts for the effectiveness of inhaled insulin in patients diagnosed with type 1 diabetes.  

In 2006, the Food and Drug Administration (FDA) approve the Exubera. However, Exubera was available in the market for only one year and was later withdrawn from the market by Pfizer [28]. In 2014, FDA approve technosphere insulin as an alternative to subcutaneous bolus insulin. The technosphere was quicker than subcutaneous insulin with a shorter duration of action [29]. The most recently published RCT evaluated the effectiveness of technosphere insulin with insulin lispro, and found that HbA1c was unchanged in both groups, however, the technosphere insulin was associated with improved post-meal glucose and a lower risk of hypoglycemia in a 16-week period [25]. Similar results were reported by another recent study conducted by Seaquist et al., who found technosphere was associated with the lesser hypoglycemic event than subcutaneous insulin aspart in 24 weeks [24].  

Except for Skyler et al., all the included studies showed short-term effectiveness of inhaled insulin in comparison to subcutaneous insulin (≤ 24 weeks). Skyler et al., conducted a three-year-long RCT, to assess the pulmonary safety of Exubera during discontinuation and administration. They found improvement in forced expiratory volume in one second (FEV1) and carbon monoxide diffusing capacity (DLco), the changes were consistent with reversible, non-progressive, and non-pathological effects on the lung. They found an increase in median insulin antibody in the Exubera group, while a reduction of antibody in the subcutaneous group, however, the antibody did not correlate with HbA1c, hypoglycemia, and lung functioning. Therefore, the inhaled insulin was better in terms of weight gain and equally effective in glycemic control in long-term effects. The insulin reduces glycosuria and its caloric expenditures, and stimulates the fatty acid accumulation in adipose tissues, favoring an increase in adipose mass [18]. Weight gain is a common concern of patients with diabetes [18]. The current evidence showed a significant improvement in weight with inhaled insulin in comparison to subcutaneous insulin.  

The cough was the most pronounced adverse effect of inhaled insulin. Chang et al. reported that cough is the common adverse event of inhaled insulin, the high prevalence of cough is related to the complex formulation containing cough inducers such as mannitol, sodium citrate, glycine, and sodium hydroxide, however, the rate of coughing was more common in exubera than in technosphere [30]. Other reported adverse effects were dyspnea and hypoglycemia. Comulada et al., reported one case of allergic alveolitis [20]. However, more concerning adverse effect was raised in insulin antibodies and lung function. Skyler et al., found no correlation of raised antibodies with blood glucose level and lung function, in a 3-year long RCT. They also found no pulmonary pathology associated with inhaled insulin. The results of the analysis showed a non-significant association of adverse events between subcutaneous and inhaled insulin [18].  

Therefore, inhaled insulin can be used as an alternative to subcutaneous insulin in patients not compliant with subcutaneous injections. It can also be used in patients concerned about weight gain with conventional insulin. The inhaled insulin is associated with a similar effect on blood glucose levels with fewer hypoglycemic shifts. Further long-term studies should be conducted on this subject to evaluate a better understanding of the safety and efficacy of inhaled insulin. 

Limitations 

Our study was limited by the following factors: (a) Asian and European regions were not appropriately covered in our analysis; (b) there was high heterogeneity seen between the studies; (c) children and adolescent age group was not covered adequately. These studies were pivotal in forming analysis, but more studies with the community and random controls should be conducted. 

## Conclusions

In this systematic review and meta-analysis, we found that inhaled insulin is equally effective as subcutaneously administered insulin in patients with diabetes type 1. The inhaled insulin was found to show less weight gain and fewer hypoglycemic shifts, with a similar effect on the blood glucose level. No significant difference was observed in the incidence of adverse events. Cough was the most pronounced adverse effect of inhaled insulin. Also, our results suggest that inhaled insulin combined with other baseline insulin gives better results than isolated treatment with baseline insulins. Inhaled insulin has a very short duration of action, therefore, should not be used by itself but in combination with other basal insulins. We suggest the use of inhaled insulin in patients not compliant with injective insulin or patients concerned about weight gain. 
